# Ethylene modulates the phenylpropanoid pathway by enhancing *VvMYB14* expression via the ERF5-melatonin-ERF104 pathway in grape seeds

**DOI:** 10.1093/hr/uhaf061

**Published:** 2025-02-25

**Authors:** Shiwei Gao, Fei Wang, Shengnan Wang, Jiapeng Diao, Shuxia Lan, Yujiao Xu, Xinning Lyu, Hui Kang, Yuxin Yao

**Affiliations:** Key Laboratory of Biology and Genetic Improvement of Horticultural Crops in Huang-Huai Region, Ministry of Agriculture, College of Horticulture Science and Engineering, Shandong Agricultural University, No.61 Daizong Street, Tai-An 271018, Shandong, China; Key Laboratory of Biology and Genetic Improvement of Horticultural Crops in Huang-Huai Region, Ministry of Agriculture, College of Horticulture Science and Engineering, Shandong Agricultural University, No.61 Daizong Street, Tai-An 271018, Shandong, China; Key Laboratory of Biology and Genetic Improvement of Horticultural Crops in Huang-Huai Region, Ministry of Agriculture, College of Horticulture Science and Engineering, Shandong Agricultural University, No.61 Daizong Street, Tai-An 271018, Shandong, China; Key Laboratory of Biology and Genetic Improvement of Horticultural Crops in Huang-Huai Region, Ministry of Agriculture, College of Horticulture Science and Engineering, Shandong Agricultural University, No.61 Daizong Street, Tai-An 271018, Shandong, China; Key Laboratory of Biology and Genetic Improvement of Horticultural Crops in Huang-Huai Region, Ministry of Agriculture, College of Horticulture Science and Engineering, Shandong Agricultural University, No.61 Daizong Street, Tai-An 271018, Shandong, China; Key Laboratory of Biology and Genetic Improvement of Horticultural Crops in Huang-Huai Region, Ministry of Agriculture, College of Horticulture Science and Engineering, Shandong Agricultural University, No.61 Daizong Street, Tai-An 271018, Shandong, China; Key Laboratory of Biology and Genetic Improvement of Horticultural Crops in Huang-Huai Region, Ministry of Agriculture, College of Horticulture Science and Engineering, Shandong Agricultural University, No.61 Daizong Street, Tai-An 271018, Shandong, China; Key Laboratory of Biology and Genetic Improvement of Horticultural Crops in Huang-Huai Region, Ministry of Agriculture, College of Horticulture Science and Engineering, Shandong Agricultural University, No.61 Daizong Street, Tai-An 271018, Shandong, China; Key Laboratory of Biology and Genetic Improvement of Horticultural Crops in Huang-Huai Region, Ministry of Agriculture, College of Horticulture Science and Engineering, Shandong Agricultural University, No.61 Daizong Street, Tai-An 271018, Shandong, China

## Abstract

The interaction between ethylene and melatonin in the regulation of polyphenol metabolism and the underlying mechanism remain largely unclear. This work demonstrated that ethylene treatment increased melatonin biosynthesis by inducing the *VvASMT* expression in grape seeds. Ethylene-induced VvERF5 transactivated *VvASMT* via binding to the ethylene response element in its promoter. *VvERF5* overexpression led to an increase in melatonin biosynthesis while its suppression generated the opposite results in grape seeds, calli, and/or *Arabidopsis* seeds. A melatonin-responsive element (MTRE) was identified, and melatonin-induced VvERF104 was found to bind to the MTRE of the *VvMYB14* promoter and activate its expression. *VvMYB14* overexpression widely modified the expression of genes in the phenylpropanoid pathway and phenolic compound content in grape seeds. DNA affinity purification sequencing revealed that the MEME-1 motif was the most likely binding sites of *VvMYB14*. *VvPAL*, *VvC4H*, and *VvCHS* were verified to be the target genes of VvMYB14. Additionally, the overexpression of *VvERF5* or *VvERF104* increased the expression of *VvPAL*, *VvC4H*, and *VvCHS*, as well as the levels of the corresponding metabolites. Moreover, the roles of *VvERF5*, *VvASMT*, and *VvERF104* in mediating ethylene-induced changes in the phenylpropanoid pathway were elucidated using their suppressing seeds. Collectively, ethylene increased the *VvMYB14* expression via the pathway of ERF5-melatonin-ERF104 and thereby modified the phenylpropanoid pathway.

## Introduction

Grape berries are rich in phenolic compounds, including flavonoids such as anthocyanins and proanthocyanidins, as well as nonflavonoid compounds such as phenolic acids and stilbenes [1]. These phenolics not only substantially affect the flavor of red wine but also act as potent antioxidants, offering potential health benefits. Approximately 30% of the total phenolics in grapes are stored in the seeds [2], making grape seeds an ideal tissue for studying phenolic compound metabolism. Ethylene and melatonin are two crucial signaling molecules involved in grape berry ripening [3], and they also play a role in regulating phenolic compound metabolism in grape berries [4, 5]. It has been reported that melatonin accumulates continuously in seeds during grape ripening, and treatment with melatonin induces proanthocyanin biosynthesis in grape seeds [6]. VvERF104 activates the expression of *VvMYBPA2* and enhances proanthocyanidin synthesis in grape seeds, suggesting a potential role for ethylene in increasing proanthocyanidin accumulation [6]. However, the specific roles of ethylene and melatonin in regulating polyphenol metabolism in seeds remain largely unknown.

The role of ethylene in regulating polyphenol metabolism has been reported in other crops. For instance, increased levels of total phenolics and *p*-coumaric acid have been observed in ethylene-treated banana fruits [7]. Ethylene application can also effectively reduce lignin content while increasing the secondary metabolites in ramie [8]. Ethylene response transcription factors (ERFs) direct specific responses to ethylene signaling by binding directly to promoter regions of ethylene-responsive genes, thereby regulating their expression [9]. In addition, ERFs play a crucial role in regulating fruit ripening, including polyphenol metabolism [10]. For example, *SmERF1L1* regulates the biosynthesis of tanshinones and phenolic acids in *Salvia miltiorrhiza* [11]. Ethylene also interacts with other hormones, including gibberellin (GA), abscisic acid (ABA), indole-3-acetic acid (IAA), and melatonin [12–14]. ERFs, such as tomato *SlERF.B3* and pear *PuERF2*, also participate in this interaction [13, 14]. While the interaction between ethylene and melatonin has been reported [4, 15], further investigation is needed to demonstrate the role of their interaction in regulating the phenylpropanoid pathway and the underlying molecular mechanisms.

Melatonin is an indolic compound derived from serotonin (5-hydroxytryptamine), and its synthesis in plants is catalyzed mainly by six enzymes, namely, tryptophan decarboxylase (TDC), tryptophan hydroxylase (TPH), tryptamine 5-hydroxylase (T5H), serotonin *N*-acetyltransferase (SNAT), *N*-acetylserotonin methyltransferase (ASMT), and caffeic acid *O*-methyltransferase (COMT) [4, 15]. Among these, ASMT/COMT and SNAT directly catalyze melatonin synthesis [16]. It is still unclear whether the key genes responsible for melatonin synthesis can respond to ethylene and participate in the interaction between ethylene and melatonin. Melatonin functions not only as a potent antioxidant but also as a multifunctional signaling molecule [17]. Melatonin treatment has been shown to increase the levels of phenolic compounds in goji berries [18]. Although melatonin signaling in plants remains largely unexplored, it was reported to interact with other signaling molecules, including H_2_O_2_, nitric oxide (NO), and various hormones [19]. In particular, melatonin promoted grape berry ripening by increasing the levels of ABA, H_2_O_2_, and ethylene [3]. Plant cell signaling is partially dependent on transcriptional regulatory networks, which consist of circuits of transcription factors and regulatory DNA elements that control the expression of target genes [20]. Thus, identifying key transcription factors and DNA elements involved in melatonin-regulated processes can provide insights into the underlying mechanisms.

MYB14 is a key transcription factor that participates in the interaction between ethylene and melatonin [4, 6]. It also plays a vital role in regulating the phenylpropanoid pathway. In grapes *(Vitis vinifera)*, VvMYB14 regulates stilbene biosynthesis by transactivating *VvSTS* expression [21, 22]. In addition, MYB14 regulates other secondary metabolites derived from the phenylpropanoid pathway. For instance, *VvMYB14* affects the accumulation of secondary metabolites in grape calli [4] and enhances proanthocyanidin biosynthesis by upregulating the expression of *VvMYBPA1* and *VvMYBPA2* in grape seeds [6]. Overexpression of *MtMYB14* was reported to strongly induce proanthocyanidin and mucilage biosynthesis in the seeds and hairy roots of *Medicago truncatula* [23]. Moreover, MYB14 could increase condensed tannin levels in *Trifolium repens* [24]. These findings suggest that MYB14 regulates multiple reactions within the phenylpropanoid pathway. Identifying the specific compounds and target genes regulated by MYB14 will contribute to a better understanding of its functions. Furthermore, *MYB14* is known to be involved in melatonin signaling because it is strongly induced by melatonin [6]. However, the signal cascade from melatonin to MYB14 remains unclear.

Phenolics are primarily synthesized through the phenylpropanoid pathway. MYB14 regulates the phenylpropanoid pathway; however, its specific target genes are yet to be identified. In addition, the mechanisms through which ethylene and melatonin regulate the phenylpropanoid pathway via MYB14 remain unclear. The present study investigated the function of *VvMYB14* in regulating the phenylpropanoid pathway by identifying its target genes in grape seeds and elucidated the ERF5-melatonin-ERF104 pathway, which mediates ethylene-induced expression of *VvMYB14*. These findings provide new insights into the molecular mechanisms underlying the role of ethylene signaling in the regulation of melatonin and phenylpropanoid biosynthesis in grape seeds.

## Results

### Ethylene increases melatonin synthesis via VvERF5-induced *VvASMT* expression

**Figure 1:**
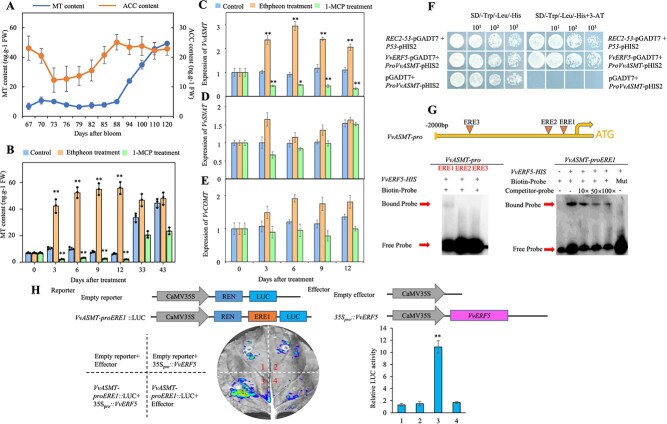
Screening of ethylene-induced melatonin synthesis-related genes and their upstream transcription factors. (**A**) Changes in ACC and melatonin content during grape seed ripening. (**B–E**) Effects of ethephon and 1-MCP treatments on melatonin content and the expression of *VvASMT*, *VvSNAT*, and *VvCOMT* in grape seeds. (**F**) Y1H assay showing the binding of *VvERF5* to ERE1 in the *VvASMT* promoter. (**G**) EMSAs demonstrating the binding of *VvERF5* to ERE1, ERE2, and ERE3 within the *VvASMT* promoter. (**H**) Fluorescence observations from the Dual-LUC assay and relative LUC activity measurements. The data are presented as the means ± SDs of three replicates in (A–F). ^*^, significant difference, *P* < 0.05; ^**^, highly significant difference, *P* < 0.01; MT, melatonin. The sequences of ERE1, ERE2, ERE3, and mERE1 are listed in Table S1.

To explore the relationship between ethylene and melatonin during grape seed ripening, changes in ACC (a precursor of ethylene synthesis) and melatonin content were monitored. The ACC content decreased sharply from the first sampling time point and reached the minimum at 73 DAB. Subsequently, the ACC content continuously increased, peaking at 88 DAB and remaining at a high level in the late stages. By contrast, melatonin levels began to rise steadily from 88 DAB, reaching their maximum in ripened seeds (Fig. 1A). This finding indicates that the peak in ACC accumulation occurred earlier than the increase in melatonin content. Furthermore, exogenous treatment with ethephon increased the melatonin accumulation, whereas treatment with 1-MCP reduced the melatonin content in seeds from 3 to 12 days after treatment (DAT; Fig. 1B). Ethephon treatment also upregulated the expression of the melatonin synthesis-related gene *VvASMT*, whereas 1-MCP led to the opposite effect. However, neither ethephon treatment nor 1-MCP treatment led to significant changes in the expression of other melatonin synthesis-related genes, *VvSNAT* and *VvCOMT* (Fig. 1C–E). Additionally, similar changing patterns were also found between melatonin accumulation and *VvASMT* expression (Fig. 1A and Fig. S1). These findings suggest that ethylene promotes melatonin synthesis by increasing *VvASMT* expression.

To identify upstream transcription factors involved in this regulation, a Y1H screening was conducted using ERE1 in the *VvASMT* promoter. This screening identified VvERF5, VvERF016, and VvERF017 as candidate transcription factors. Further analysis revealed that ethephon treatment substantially increased the expression of corresponding genes in grape seeds, whereas 1-MCP treatment led to a decrease (Fig. S2). *VvERF5* exhibited a similar expression pattern to *VvASMT* in seeds from 73 to 120 DAB (Fig. S1). Subsequent experiments, including Y1H, EMSA, and Dual-LUC assays, demonstrated that *VvERF5* specifically bound to ERE1 in the *VvASMT* promoter (Fig. 1F–H) but did not bind to ERE2 or ERE3 (Fig. 1G). In the LUC assays, tobacco leaves cotransformed with the ERE1-35S mini-LUC reporter and the 35S-*VvERF5* effector showed a marked increase in the fluorescence intensity and relative LUC activity, confirming the positive role of *VvERF5* in regulating *VvASMT* expression (Fig. 1H).

**Figure 2:**
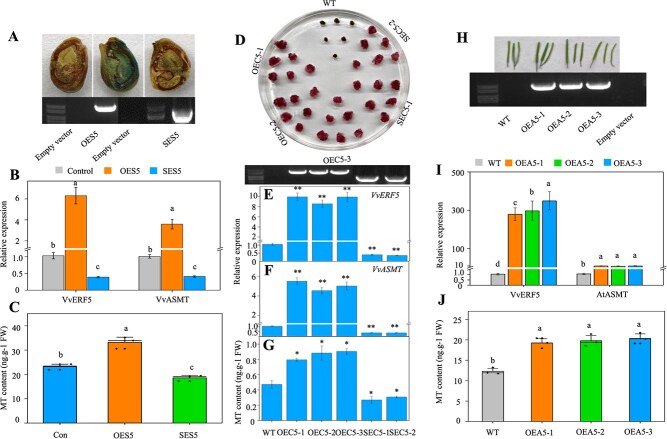
Characterization of the role of *VvERF5* in regulating melatonin synthesis through *VvASMT* expression. (**A, B**) Identification of seeds with transient overexpression (OES5) and suppression (SES5) of *VvERF5* using PCR (**A**) and qPCR (**B**) and corresponding changes in *VvASMT* expression. (**A**) shows the construct 35S::ERF5-GUS used to generate seeds overexpressing *VvERF5-GUS*, with GUS staining used for validating the method’s feasibility. (**C**) Melatonin content in the control, OES5, and SES5 seeds. (**D**) Screening of grape calli overexpressing *VvERF5* and those with suppressed expression using a selective medium. The photograph was taken 25 days after subculture in the selective medium. (**E**) Identification of *VvERF5*-overexpressing and -suppressing calli using PCR and qPCR. (**F, G**) Expression levels of *VvASMT* (**F**) and melatonin content (**G**) in WT and transgenic calli. (**H**) PCR identification of *VvERF5*-overexpressing *Arabidopsis* plants. (**I, J**) Expression levels of *VvERF5* and *AtASMT* (**I**), and melatonin accumulation (**J**) in *Arabidopsis* seeds. The values represent the means ± SD of three replicates. ^*^, Significant difference, *P* < 0.05; ^**^, highly significant difference, *P* < 0.01. The values indicated by the different lowercase letters are significant at *P* < 0.05; MT, melatonin. The primers used are listed in Table S1.

To further investigate the role of *VvERF5* in promoting melatonin synthesis, grape seeds were engineered to overexpress *VvERF5* or exhibit suppressed *VvERF5* expression (Fig. 2A and B). Overexpression of *VvERF5* significantly increased both *VvASMT* expression and melatonin content in seeds, whereas suppression of *VvERF5* expression led to the opposite effects (Fig. 2B and C). In addition, three lines of *VvERF5*-overexpressing grape calli (OEC5-1, -2, and -3) and two lines with suppressed *VvERF5* expression (SEC5-1 and -2) were generated (Fig. 2D and E). Consistent with the results in seeds, *VvASMT* expression and melatonin content were significantly increased in calli with gene overexpression and decreased in those with suppressed expression (Fig. 2E–G). The role of *VvERF5* in enhancing *AtASMT* expression and melatonin content was also validated in three lines of *VvERF5*-overexpressing *Arabidopsis* plants (Fig. 2H–J).

In summary, ethylene induced the expression of *VvERF5*, which, in turn, transactivated *VvASMT*, leading to increased melatonin biosynthesis.

### Identification of the melatonin-responsive element in the *VvMYB14* promoter

**Figure 3:**
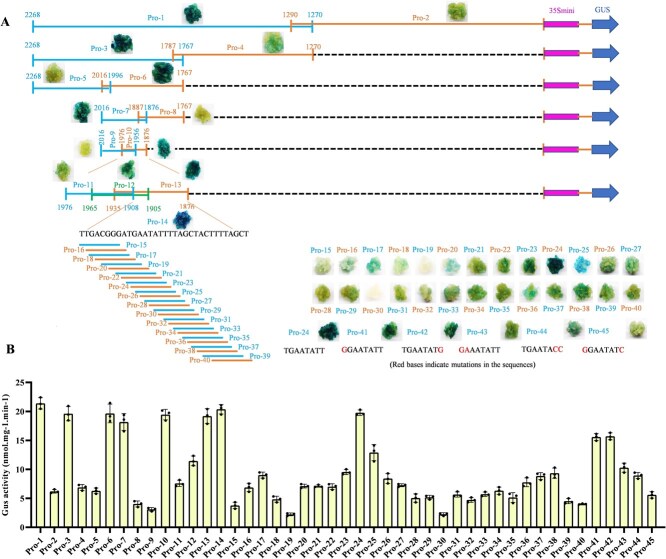
GUS staining (**A**) and activity assays (**B**) of grape calli expressing the *VvMYB14* promoter fragment-35S mini-GUS constructs. In (**A**), different *VvMYB14* promoter fragments are represented by different colored solid lines, with the number indicating the fragment length. Panel (**B**) depicts GUS activity corresponding to these promoter fragments.

Our previous study demonstrated that the gene *VvMYB14* is strongly induced by melatonin in grape seeds [6]. In this study, we selected a 2268-bp region upstream of the ATG start codon as the *VvMYB14* promoter to identify potential melatonin-responsive elements (MTREs). The promoter was divided into 40 fragments, labeled Pro-1 through Pro-40, which were then inserted into an expression vector to create constructs of each Pro fragment fused to a 35S mini-GUS reporter gene (Fig. 3A). These constructs were transformed into grape calli to evaluate their response to melatonin through GUS staining and GUS activity assays (Fig. 3B). Initially, Pro-1 was found to be induced by melatonin, as indicated by its more intense blue color and higher GUS activity compared with Pro-2. Subsequently, Pro-1 was further divided into two smaller fragments, Pro-3 and Pro-4, with Pro-3 showing strong induction by melatonin. Following this method, a 33-bp fragment, designated Pro-14, was identified as highly responsive to melatonin. Pro-14 was then further divided into 25 shorter fragments, each 8 bp in length. Among these, Pro-24 exhibited the strongest GUS staining intensity and highest activity. By contrast, mutations in 1 or2 bp within Pro-24 significantly reduced GUS staining and activity (Fig. 3). Collectively, these results identified Pro-24 (sequence: TGAATATT) as a key MTRE within the *VvMYB14* promoter.

### Melatonin-induced VvERF104 binds to the melatonin-responsive element in the *VvMYB14* promoter and increases its expression

**Figure 4:**
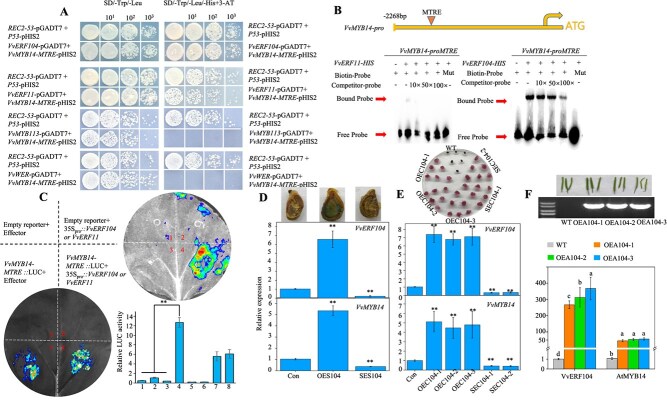
Characterization of the role of VvERF104 in increasing *VvMYB14* expression by binding to the MTRE. (**A**) Y1H assay demonstrating the binding of *VvERF104* and *VvERF11* proteins to the *VvMYB14* promoter. (**B**) EMSAs showing the binding of VvERF104 and VvERF11 proteins to the MTREs. (**C**) Representative images of tobacco leaves at 60 h after infiltration, showing corresponding LUC activity. The upper and lower images correspond to the LUC assays of *VvERF104* and *VvERF11*, respectively. (**D**) Identification of seeds with transient overexpression (OES104) and suppression (SES104) of *VvERF104* using qPCR and/or GUS staining, along with changes in *VvMYB14* expression in the transgenic seeds. (**E**) Identification of *VvERF104*-overexpressing and -suppressing calli using 15 mg·L^−1^ hygromycin selection medium and qPCR, along with changes in *VvMYB14* expression in the transgenic calli. (**F**) Identification of *VvERF104*-overexpressing *Arabidopsis* plants using PCR and qPCR, along with changes in *AtMYB14* expression in the transgenic plants. The values represent the means ± SD of three replicates. ^**^, highly significant difference, *P* < 0.01. The sequences of MTRE and mMTRE are listed in Table S1.

The *VvMYB14* promoter fragment containing the MTRE was used as bait in a Y1H screening to identify interacting transcription factors. Four transcription factors—VvMYB113, VvWER, VvERF104, and VvERF11—were identified as potential candidates. Among these, all except *VvWER* were significantly induced by melatonin in grape seeds (Fig. S3). Y1H assays further demonstrated that VvERF104 and VvERF11 directly bound to the MTRE within the *VvMYB14* promoter (Fig. 4A). The binding of VvERF104 to the MTRE was confirmed through EMSA and Dual-LUC assays. In these assays, *VvERF104* significantly increased the expression of the LUC reporter gene, as indicated by higher luminescence intensity and relative LUC activity. By contrast, VvERF11 showed weak binding to the MTRE, as evidenced by a faint band in the EMSA and comparable luminescence intensity and relative LUC activity to controls (Fig. 4B and C). Furthermore, overexpression of *VvERF104* in grape seeds led to a significant increase in *VvMYB14* expression, whereas suppression of *VvERF104* resulted in decreased *VvMYB14* expression (Fig. 4D). Similar results were observed in *VvERF104*-overexpression and -suppression grape calli (Fig. 4E). Moreover, overexpression of *VvERF104* in three transgenic *Arabidopsis* lines significantly enhanced the expression of *AtMYB14* in *Arabidopsis* seeds (Fig. 4F). In summary, melatonin induced the expression of *VvERF104*, which, in turn, transactivated *VvMYB14* by binding to the MTRE in its promoter.

### *VvMYB14* overexpression widely regulates gene expression and metabolite accumulation in the phenylpropanoid pathway

**Figure 5:**
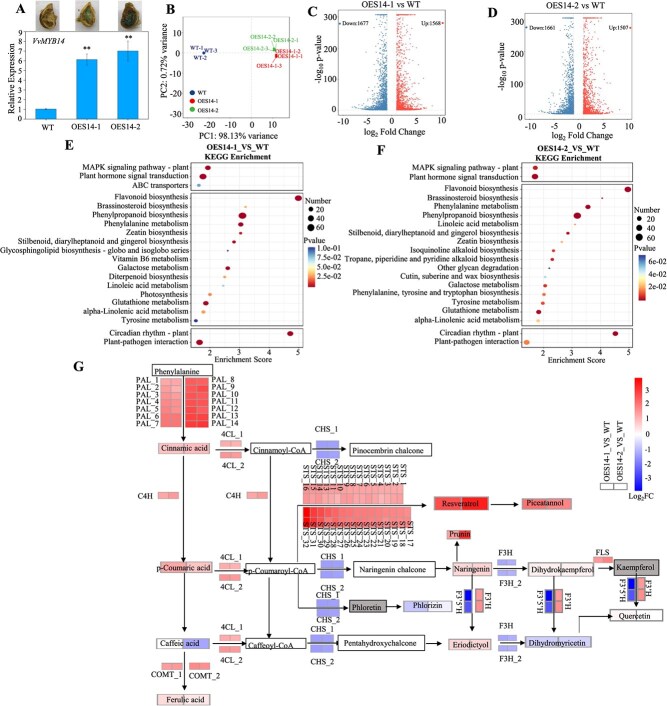
Changes in gene expression and metabolite content in the phenylpropanoid pathway caused by *VvMYB14* overexpression in grape seeds. (**A**) Identification of transient overexpression of *VvMYB14* in grape seeds using GUS staining and qPCR. (**B**) PCA of the nine samples based on FPKM values. (**C, D**) Volcano plots showing upregulated and downregulated genes in the comparison groups OES14-1 vs WT (**C**) and OES14-2 vs WT (**D**). (**E, F**) KEGG enrichment analysis of DEGs in OES14-1 (**E**) and OES14-2 (**F**) compared to WT. (**G**) KEGG pathway analysis of metabolites and genes whose accumulation and expression, respectively, were altered in the OE14 seeds compared with the WT seeds. Each solid black arrow represents an enzyme-catalyzed process. White background boxes indicate metabolites not detected in this study, gray boxes represent undetectable metabolites, and colored boxes represent metabolites and DEGs with altered accumulation and expression due to *VvMYB14* overexpression.

To investigate the function of *VvMYB14*, two groups of *VvMYB14*-overexpressing grape seeds (OES14-1 and OES14-2) were generated (Fig. 5A). RNA-Seq was performed on WT, OES14-1, and OES14-2 seeds to evaluate the gene expression changes resulting from *VvMYB14* overexpression. Principal component analysis (PCA) of the nine samples revealed that the first principal component (PC1) accounted for 98.13% of the variance, indicating a substantial difference between WT and *VvMYB14*-overexpressing seeds (Fig. 5B). A total of 3245 and 3168 DEGs were identified in the comparisons of WT vs OES14-1 and WT vs OES14-2, respectively (Fig. 5C and D; Tables S2 and S3). KEGG enrichment analysis showed that the DEGs were primarily associated with flavonoid biosynthesis, followed by phenylpropanoid biosynthesis, phenylalanine metabolism, and plant hormone signal transduction pathways (Fig. 5E and F).

Targeted metabolomics was conducted to identify DAMs, which revealed a total of 130 phenolic compounds; among these, 70 compounds, including 52 phenolic compounds, were present in grape seeds (Table S4). Compared with WT seeds, 17 phenolic compounds were found to be more abundant, whereas 14 were less abundant in the *VvMYB14*-overexpressing seeds (Table S4). An association analysis of DAMs and DEGs revealed that the significant upregulation of 14 phenylalanine ammonia-lyases (PALs), one cinnamate-4-hydroxylase (C4H), and two caffeic acid *o*-methyltransferases (COMTs) dominated the phenylpropanoid biosynthesis pathway, leading to increased levels of cinnamic acid, *p*-coumaric acid, and ferulic acid (Fig. 5G). Notably, the expression of 32 stilbene synthase genes significantly increased, which corresponded with elevated levels of resveratrol and piceatannol, leading to increased stilbene biosynthesis.

### VvMYB14 directly binds to the promoters of *VvPAL, VvC4H*, and *VvCHS* and regulates their expression

**Figure 6:**
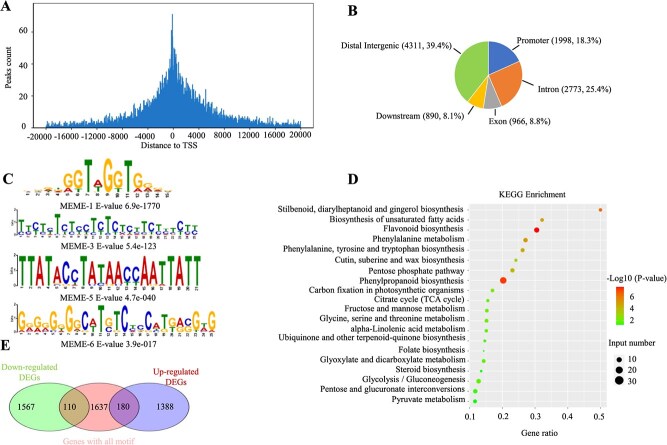
Genome-wide identification of *VvMYB14* binding sites using DAP-seq. (**A**) Distribution of *VvMYB14* binding sites within the −20 000 to +20 000-bp region flanking the TSS. (**B**) Number and percentage of peaks in different genomic regions. (**C**) Predicted *VvMYB14* binding motif with a high enrichment score. (**D**) KEGG analysis of putative target genes of *VvMYB14*. (**E**) Venn diagram showing the overlapping genes identified through DAP-seq and RNA-seq.

DAP-seq was conducted to identify *VvMYB14* binding sites across the grape genome. A total of 10 939 peaks, which were included in 8769 genes, were uncovered (Table S5). The binding peaks were predominantly located near the transcription start sites (TSSs) of target genes (Fig. 6A), with 18.3% of the peaks found within promoter regions (up to 2.0 kb upstream of the TSS) (Fig. 6B). A total of 1998 high-confidence VvMYB14 binding sites were discovered, which were located in the promoter regions of 1932 putative target genes (Table S5). Based on the 29 836 motifs in the promoter regions of 1927 unique genes (Table S6), the significantly enriched motifs (*E*-value <0.05) were identified using MEME-chip. It was indicated that the MEME-1 motif (DDDDGGTWGGTGRRD) had the highest enrich score, followed by MEME-3 (TTYTYTCTYCTYTCTCTTCTYTCTY), MEME-5 (TTATACCTATAACCAATTATT), and MEME-6 (GRGRGKGGCWTSTCYCCATKRSGWG) (Fig. 6C). KEGG analysis revealed that the putative target genes of *VvMYB14* were highly enriched in pathways related to phenylpropanoid biosynthesis; flavonoid biosynthesis; and stilbenoid, diarylheptanoid, and gingerol biosyntheses (Fig. 6D).

**Figure 7:**
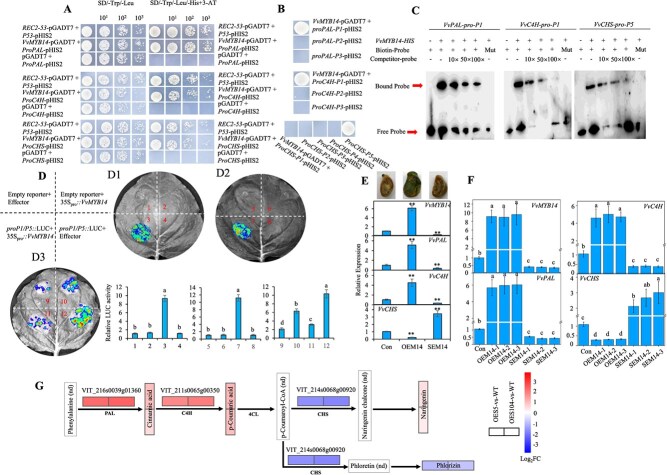
Identification of *VvMYB14* target genes in the phenylpropanoid pathway. (**A**) Y1H assays showing the binding of *VvMYB14* to the promoters of *VvPAL*, *VvC4H*, and *VvCHS*, all containing the putative binding sites. The yeast cells diluted 1-, 10-, 100-, and 1000-fold are shown from left to right. (**B**) Y1H assays showing the binding of *VvMYB14* to specific sites in the promoters of *VvPAL*, *VvC4H*, and *VvCHS*. **(C**) EMSAs showing the binding of VvMYB14 to *cis*-regulatory elements in the *VvPAL*, *VvC4H*, and *VvCHS* promoters. (**D**) Dual-LUC assay and LUC activity in tobacco leaves 60 h after infiltration. D1, D2, and D3 indicate the binding of VvMYB14 to the P1/P5 sites in the promoters of *VvPAL*, *VvC4H*, and *VvCHS*, respectively. (**E, F**) Identification of *VvMYB14*-overexpressing and -suppressing seeds (**E**) and calli (**F**) using GUS staining and/or qRT-PCR, and the effects of altered *VvMYB14* expression on *VvPAL*, *VvC4H*, and *VvCHS* expressions. (**G**) Changes in the content of metabolites and the expression of genes involved in phenylpropanoid pathway. The colored boxes represent metabolites and DEGs with altered accumulation and expression due to *VvERF5* and *VvERF104* overexpression. nd means not detected. The values represent the means ± SD of three replicates. ^**^, highly significant difference, *P* < 0.01. The values indicated by the different lowercase letters are significant at *P* < 0.05.

A combined analysis of DAP-seq and RNA-seq data revealed that 180 upregulated and 110 downregulated DEGs contained *VvMYB14*-binding motifs (Fig. 6E; Table S7). Among these, 78 DEGs were related to phenylpropanoid and flavonoid biosynthesis (Table S7). From this group, 10 DEGs involved in the phenylpropanoid pathway and containing the MEME-1 motif were selected for Y1H assays (Fig. 7A; Fig. S4). Y1H assays demonstrated that *VvMYB14* directly bound to P1 sites in the promoters of *VvPAL* and *VvC4H*, as well as to the P5 site in the promoter of *VvCHS*, with all these sites containing the MEME-1 motif (Fig. 7A and B; Table S1). Further validation using EMSA and LUC analysis confirmed that *VvMYB14* was bound to the promoters of *VvPAL*, *VvC4H*, and *VvCHS* (Fig. 7C and D). LUC analysis also revealed that *VvMYB14* increased LUC expression by binding to the P1 site of the *VvPAL* or *VvC4H* promoter and reduced LUC expression by binding to the P5 site of the *VvCHS* promoter (Fig. 7D). qRT-PCR showed that the overexpression of *VvMYB14* led to increased expression of *VvPAL* and *VvC4H* and decreased expression of *VvCHS* in grape seeds, whereas suppression of *VvMYB14* produced the opposite effects (Fig. 7E). Similar results were observed in *VvMYB14*-overexpressing grape calli and those with suppressed expression (Fig. 7F). In summary, these findings indicated that *VvMYB14* regulated the transcription of *VvPAL*, *VvC4H*, and *VvCHS* by binding to the MEME-1 motif in their promoters.

### ERF5-melatonin-ERF104 pathway participates in ethylene-induced expression of the genes involved in the phenylpropanoid pathway

Since *VvERF5* regulated melatonin synthesis (Fig. 2), melatonin-induced *VvERF104* expression (Fig. S3), and VvERF104 transactivated *VvMYB14* (Fig. 4), we proposed the ERF5-melatonin-ERF104 pathway that regulated the expression of *VvMYB14*. Additionally, overexpression of *VvERF5* and *VvERF104* modified the expression of *VvPAL*, *VvC4H*, and *VvCHS*, as well as the contents of cinnamic acid and three other metabolites (Fig. 7G). Therefore, the ERF5-melatonin-ERF104 pathway enhanced *VvMYB14* expression and modified the phenylpropanoid pathway. Furthermore, treatments with ethylene and melatonin significantly altered the expression of several key genes, including *VvERF5*, *VvASMT*, *VvERF104*, *VvMYB14*, *VvPAL*, *VvC4H*, and *VvCHS,* in grape seeds (Fig. S5). By contrast, suppression of *VvERF5*, *VvASMT*, or *VvERF104* mitigated the ethylene-induced increases in the expression of *VvMYB14*, *VvPAL*, and *VvC4H* while enhancing the ethylene-induced expression of *VvCHS*. These results underscored the critical role of these genes in ethylene signaling. Moreover, the suppression of *VvASMT* reduced the ethylene-induced expression of these genes, indicating that ethylene’s effects are partially mediated through melatonin. Based on these findings, the regulatory pathway of ERF5-melatonin-ERF104 was proposed to participate in the regulation of ethylene on the phenylpropanoid pathway.

## Discussion

### VvERF5 might regulate the phenylpropanoid pathway via multiple pathways including melatonin signaling

Grapes, as a typical nonclimacteric fruit, do not typically exhibit a peak in ethylene release during ripening. However, ethylene release peaks have been detected in grape varieties, such as Cabernet Sauvignon, Moldova, and Muscat Hamburg [3, 25, 26]. In this study, an ACC accumulate peak was observed in Merlot grape seeds at 88 DAB (Fig. 1A). This suggests that significant ethylene production occurs in various grape tissues before the onset of ripening, which precedes the peak in melatonin accumulation during berry ripening (Fig. 1A; 20). Furthermore, ethylene treatment was found to increase melatonin synthesis, whereas treatment with 1- MCP produced the opposite effect (Fig. 1B). These findings suggest that ethylene may trigger or at least modulate melatonin synthesis during fruit development.

ERFs are downstream components of the ethylene signaling pathway that regulate the expression of ethylene-responsive genes by directly binding to their promoter regions [9]. In this study, *VvERF5* expression in the seeds increased after ethephon treatment but decreased with 1-MCP treatment (Fig. S2). Similar to the role of *SlERF5* in tomatoes, which induces the ethylene-responsive ‘triple response’ phenotype [27], *VvERF5* appears to play a crucial role in the ethylene signaling pathway. Our results indicated that *VvASMT* is a target gene of *VvERF5* (Fig. 1F–H). ASMT has been shown to play a rate-limiting role in melatonin synthesis in capsicum [28, 29]. This suggests that *VvERF5* regulates melatonin synthesis via *VvASMT*. This hypothesis was further supported by the effects of *VvERF5* overexpression and suppression on *VvASMT* expression and melatonin content in the seeds and calli (Fig. 2A–G). Melatonin’s role in altering the metabolism of secondary metabolites, primarily derived from the phenylpropanoid pathway, has been reported [30, 31]. Aligning with these findings, our study demonstrated that the suppression of *VvERF5* reduced ethylene-induced expression of *VvPAL* and *VvC4H* (Fig. S5), indicating that *VvERF5* regulates the phenylpropanoid pathway via melatonin signaling.

*ERF5* has been reported to regulate flavonoid biosynthesis through other pathways. In pear, *PcERF5* activates the anthocyanin synthesis–related transcription factors *PcMYB10* and *PcMYB114*, as well as *MYBA* in mulberry [32, 33]. It also transactivates flavonoid synthesis–related genes, including *DFR*, *ANS*, *UFGT*, and *F3H* [32, 33]. In addition, PcERF5 interacts with PcMYB10 to form the ERF5–MYB10 protein complex, which enhances the transcriptional activation of PcERF5 on its target genes [33]. In summary, *VvERF5* is a key component of the ethylene signaling cascade and plays a role in regulating the phenylpropanoid pathway via melatonin signaling and other mechanisms. Increasing evidence suggests that melatonin promotes ethylene biosynthesis by upregulating the expression of *ACS* and/or *ACO* in fruits, including grapes [3, 4, 34]. This finding indicates the potential existence of a regulatory circuit between ethylene and melatonin synthesis, which may contribute to the fine-tuning of the phenylpropanoid pathway.

### *VvMYB14* broadly regulates the phenylpropanoid pathway possibly by binding to different target genes

Overexpression of *VvMYB14* in grape seeds resulted in significant changes in the expression of genes involved in the phenylpropanoid pathway and altered the content of 31 phenolic compounds compared with WT seeds (Fig. 5E and F; Table S4). Specifically, a combined analysis of DAP-seq and RNA-seq revealed the presence of *VvMYB14* binding motifs in the promoter regions of 78 DEGs associated with the phenylpropanoid pathway (Fig. 6E; Table S7). Similarly, overexpression of *LiMYB14* in lotus plants increased the expression of genes involved in the general phenylpropanoid pathway, including *PAL*, *C4H*, and *4CL* [35]. These findings suggest that *MYB14* broadly modulates the phenylpropanoid pathway by regulating different target genes. Additionally, *VvPAL*, *VvC4H*, and *VvCHS* were confirmed as direct targets of *VvMYB14* (Fig. 7E and F).

PALs catalyze the first step of the phenylpropanoid pathway, converting phenylalanine into cinnamic acid [36]. Cinnamic acid serves as the initial substrate for the biosynthesis of other phenylpropanoids and phenolic compounds [37]. In this study, overexpression of *VvMYB14* led to an increase in the expression of 14 *VvPAL* genes and the content of cinnamic acid (Fig. 5G). Notably, 13 of these *VvPAL* genes also contained the MEME-1 motif in their promoters, suggesting that they might be direct targets of *VvMYB14* (Table S7). This indicates that *VvMYB14* could induce the entire phenylpropanoid pathway by enhancing the production of key initial substrates. Cinnamic acid is hydroxylated by C4H to produce *p*-coumaric acid [36], and increased expression of *VvC4H* contributed to higher levels of *p*-coumaric acid in the overexpressing seeds (Fig. 5G). Chalcone synthase (CHS) catalyzes the first committed step in flavonoid biosynthesis by directing carbon flux from general phenylpropanoid metabolism to the flavonoid pathway [38]. The downregulation of two *VvCHS* genes suggests that more substrates may be diverted to other branches, such as resveratrol biosynthesis (Fig. 5G). Ethylene treatment and *VvMYB14* overexpression had different effects on *VvCHS* expression (Fig. 7F; Fig. S5), suggesting that additional regulators are involved in controlling *VvCHS* expression within the ethylene signaling pathway.

*VvSTS41* and *VvSTS29* have been identified as target genes of VvMYB14 [21]. The significant increase in the expression of 17 *VvSTS* genes, along with higher levels of resveratrol and piceatannol (Fig. 5G), indicates the crucial role of *VvMYB14* in regulating resveratrol synthesis through the activation of *VvSTS*. Moreover, *VvMYBPA1* is directly transactivated by VvMYB14, leading to increased proanthocyanidin synthesis in grape seeds [6]. In *M. truncatula*, MtMYB14 and MtMYB5 physically interact and synergistically activate the expression of anthocyanidin reductase and leucoanthocyanidin reductase [23]. *VvMYB14* lacks the motif necessary for interaction with basic helix–loop–helix (bHLH) proteins, suggesting that *VvMYB14* induces promoter activity of target genes independently of bHLH/WD40 cofactors [6, 21], suggesting that *VvMYB14* regulates the phenylpropanoid pathway by directly controlling the expression of multiple target genes. Nevertheless, the expression of *HST, 4CL2, CCOAOMT, CCR1, COMT, CYP98A2*, and *CAD1* was largely changed by *VvMYB14* overexpression, but they were not its target genes (Tables S2 and S3), suggesting that *VvMYB14* regulates gene expression indirectly via other pathways.

### VvERF104 might integrate ethylene and melatonin signals to regulate the phenylpropanoid pathway

Ethylene and melatonin act as regulators of the phenylpropanoid pathway in grapes by modulating gene expression. In this study, both ethylene and melatonin treatments led to an increase in the expression of key genes involved in the phenylpropanoid pathway, including *VvPAL*, *VvC4H*, and *VvCHS* (Fig. S5). Similar findings have been reported in previous studies where ethylene or melatonin treatment upregulated the phenylpropanoid pathway genes, including *VvPAL*, *VvC4H*, and *VvCHS* [39, 40 ]. However, the precise mechanisms by which ethylene and melatonin regulate this pathway remain largely unknown. Here, we identified VvERF104 as a key transcription factor that is strongly induced by both ethylene and melatonin. VvERF104 was shown to bind directly to the MTRE in the promoter of *VvMYB14* (Fig. S3; Fig. 4A–C). Additionally, suppression of *VvERF104* significantly reduced the ethylene- or melatonin-induced expression of *VvMYB14*, *VvPAL*, and *VvC4H* (Fig. S5). These findings suggest that *VvERF104* plays a crucial role in integrating ethylene and melatonin signals to regulate the phenylpropanoid pathway.

The role of ERF104 in integrating ethylene and melatonin signals to induce immunity in *A. thaliana* has been previously reported. Specifically, the flg22 signaling network induces MPK6 to directly target ERF104 through phosphorylation, affecting ERF104 stability and ethylene signaling. Simultaneously, MPK3/6 and MKK4/5 stimulate ethylene production, which triggers the release of MPK6 from ERF104 in a process dependent on EIN2 and the EIN3/EIL members. The liberated ERF104 then enhances immunity by regulating its target genes, positioning ERF104 as a key regulator of basal immunity in *Arabidopsis* [41]. Melatonin has also been shown to increase the expression and phosphorylation levels of MPK3/6, which, in turn, activate several transcription factors that induce various defense genes in *Panax notoginseng* and *A. thaliana* [42, 43]. These findings suggest that melatonin may induce immunity via the MPK3/6-ERF104 pathway. It is worth investigating whether a similar mechanism exists in the regulation of phenylpropanoid metabolism by ethylene and melatonin.

In this study, VvERF104 bound to the MTRE in the promoter of *VvMYB14* and increased its expression (Fig. 4A–C). Genome-wide MTRE assays revealed that this element exists in the promoters of 90 other genes related to the phenylpropanoid pathway (Table S8). Additionally, our previous work demonstrated that *VvERF104* directly transactivates the expression of *VvMYBPA1* [6], suggesting that *VvERF104* may regulate the phenylpropanoid pathway by controlling transcription factors or structural genes, other than *VvMYB14*.

**Figure 8:**
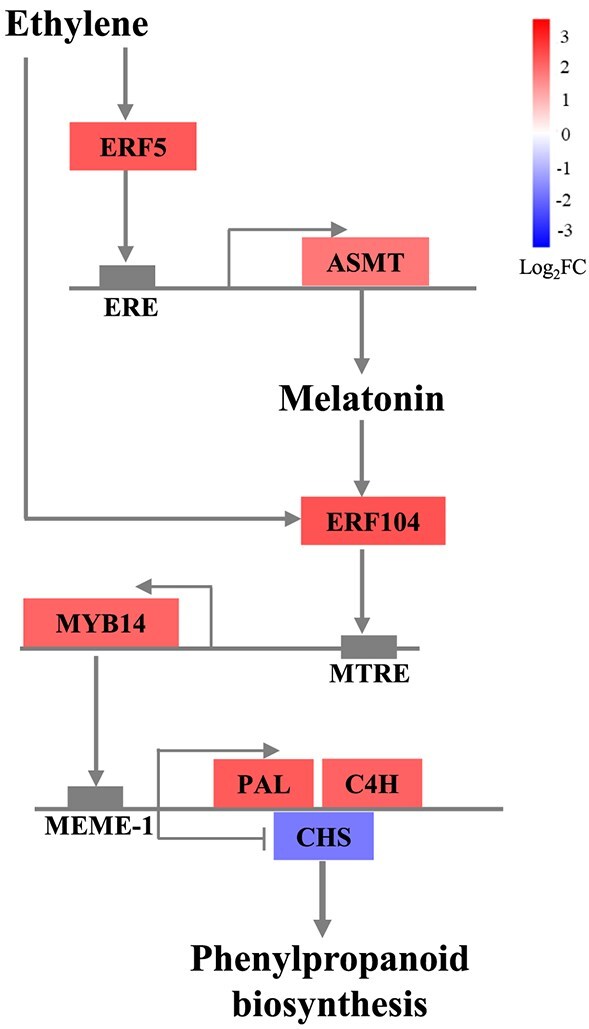
Model of phenylpropanoid pathway regulation by ethylene via the ERF5-Melatonin-ERF104 pathway. In this model, *VvERF5* transactivates *VvASMT* to increase melatonin synthesis. Ethylene promotes *VvERF104* expression directly or indirectly through melatonin. VvERF104 then binds to the MTRE in the *VvMYB14* promoter to induce its expression. *VvMYB14* regulates the expression of *VvPAL*, *VvC4H*, and *VvCHS* by binding to the MEME-1 motif.

## Conclusion

Ethylene promotes melatonin biosynthesis by inducing the expression of *VvERF5*, which transactivates *VvASMT*. Melatonin, in turn, strongly induces *VvMYB14*, which broadly modulates gene expression and metabolite content in the phenylpropanoid pathway in grape seeds. *VvMYB14* binds to the MEME-1 motif in the promoters of *VvPAL*, *VvC4H*, and *VvCHS* to regulate their expression, making it a key transcription factor in melatonin’s regulation of the phenylpropanoid pathway. Additionally, the MTRE was identified within the *VvMYB14* promoter, and *VvERF104* was shown to bind to the MTRE and activate *VvMYB14* expression. The results also indicate the roles of *VvERF5*, *VvASMT*, and *VvERF104* in mediating ethylene-induced expression of genes involved in the phenylpropanoid pathway (Fig. 8).

## Materials and methods

### Plant materials and growth conditions

Clusters of Merlot grapevines (*V. vinifera*), cultivated in an experimental vineyard in Tai-An City, Shandong Province, China, were used as experimental materials. At 67 days after bloom (DAB), grape clusters were separately treated with a solution of 250 mg·L^−1^ ethephon containing 0.05% Triton X-100 or 10 μl·L^−1^ 1-MCP. The clusters treated with 0.05% Triton X-100 alone served as the control group. In each treatment, the grape clusters were fully immersed in the respective solutions for 30 s, with three biological replicates for each treatment, and each replicate consisting of 30 clusters from five vines.

To induce nonembryogenic calli, seeds of Jianhong grapes, a new red-fleshed grape cultivar derived from the mutation of the SA15 grape, were cultured on Murashige and Skoog (MS) medium supplemented with 0.12 mg·L^−1^ indole-3-butyric acid (IBA) and 1.2 mg·L^−1^ thidiazuron (TDZ). The resulting calli were then subcultured on MS medium containing 30 g·L^−1^ sucrose, 0.60 g·L^−1^ 2-(*N*-morpholino) ethanesulfonic acid, 8 mg·L^−1^ picloram, 2.5 mg·L^−1^ TDZ, and 7 g·L^−1^ agar [6]. The calli were maintained in a growth chamber at 25°C under a 16-h light/8-h dark photoperiod.

### Determination of 1-Aminocyclopropane-1-carboxylate (ACC) and melatonin content

Extraction and determination of ACC were performed based on a previously described method [44].Melatonin was extracted and quantified following a modified version of a previously reported method [45]. Briefly, 1 g of seeds were sonicated in methanol for 20 min to extract melatonin. The supernatant was then centrifuged and evaporated to dryness at 30°C. The resulting residue was dissolved in methanol and purified using a C18 solid-phase extraction cartridge (ProElut; Dikma, China). Melatonin detection was performed using an ACQUITY ultra-high-performance liquid chromatography (UHPLC) system coupled with a quadrupole time-of-flight (QTof)-micro mass spectrometer (Waters, Milford, MA, USA). The UHPLC and MS conditions were identical to those used in our previous study [4]. Quantification of melatonin was achieved using an external calibration curve based on a melatonin standard.

### Yeast one-hybrid assays, electrophoretic mobility shift assays, and dual-luciferase assay

Yeast one-hybrid (Y1H) assays were performed following the protocol from our previous study [6]. The coding sequences (CDS) of *VvERF5*, *VvERF104*, and *VvMYB14* were cloned into the pGADT7 vector, whereas promoter fragments from the target genes were cloned into the pHIS2 vector. These resulting plasmids were transformed into the yeast strain Y187 and plated on the SD/-Trp/-Leu medium. The Y1H assay was conducted using a Y1H Library Screening Kit (Clontech, Mountain View, CA, USA) according to the manufacturer’s instructions.

For electrophoretic mobility shift assays (EMSAs), recombinant hexahistidine (HIS)-tagged *VvERF5*, *VvERF104*, and *VvMYB14* proteins were expressed using the pET-32a vector and purified with a HIS-tag purification column (Beyotime, Shanghai, China). DNA probes containing the ethylene response element (ERE), MTRE, or MEME-1 element were synthesized and labeled with biotin. EMSAs were performed according to the protocol provided in the LightShift Chemiluminescent EMSA Kit (Beyotime, Shanghai, China).

For the dual-luciferase (Dual-LUC) assay, promoter fragments of the target genes were cloned into the pGreenII 0800-LUC reporter vector, whereas the CDSs of *VvERF5*, *VvERF104*, and *VvMYB14* were inserted into the pGreenII 62-SK effector vector. Through *Agrobacterium*-mediated transformation, specified plasmid combinations were transiently introduced into tobacco leaves. The relative LUC/Renilla (REN) activity was then measured using Dual-LUC assay reagents (Promega, Wisconsin, USA).

### Stable and transient transformations of grape seeds, calli, and/or *Arabidopsis* plants

The open reading frames (ORFs) of various genes were cloned into the pRI101-glucuronidase (GUS) or pHB vectors to create constructs for overexpression, specifically 35S::VvERF5, 35S::VvERF104, and 35S::VvMYB14. For antisense suppression, the 3′-untranslated regions (3′-UTRs) of *VvERF5*, *VvASMT*, *VvERF104*, and *VvMYB14* were inserted into the pHB vector. The pRI101-GUS vectors containing ORFs were transiently transformed into grape seeds by using the *Agrobacterium tumefaciens* strain LBA4404. In this process, grape seeds were halved lengthwise, soaked in an *Agrobacterium* suspension with gentle shaking for 20 min, and then subjected to vacuum infiltration for 30 min. The seeds were cocultivated on a solid MS medium (Murashige and Skoog) containing 2% sucrose and 15 mg·L^−1^ acetosyringone at 25°C in the dark for 3 days.

For the genetic transformation of grape calli, the pHB vectors containing ORFs and 3′-UTRs were introduced for sense overexpression and antisense suppression, respectively, using *Agrobacterium*-mediated transformation [45]. Calli were immersed in an *Agrobacterium* suspension for 20 min, blotted dry, and transferred to the MS medium containing 100 μM acetosyringone. After 2 days of coculture in darkness at 25°C, the calli were screened on a B5 medium containing 250 mg·L^−1^ cefotaxime and 20 mg·L^−1^ hygromycin at 25°C.

To identify the MTRE, pCAMBIA1391 vectors with the 35S promoter replaced by different fragments of *VvMYB14* were transiently transformed into grape calli through *Agrobacterium*-mediated transformation. The calli were soaked in an *Agrobacterium* solution with gentle shaking for 20 min, followed by vacuum infiltration for 10 min. Cocultivation was performed on a solid MS medium containing 50 μM melatonin and 15 mg·L^−1^ acetosyringone for 2 days in the dark.

For *Arabidopsis* transformation, the pHB vectors containing ORFs were introduced into *Arabidopsis thaliana* Columbia-0 (Col-0) via the *Agrobacterium* strain GV3101 using the floral dip method [46].

All transgenic seeds, calli, and *Arabidopsis* plants were confirmed using β-GUS staining, polymerase chain reaction (PCR), and/or reverse transcription quantitative PCR (RT-qPCR). All primers were listed in Table S1.

### Glucuronidase staining and activity assays

Protein content was measured using a bicinchoninic acid protein assay kit (Beyotime, Shanghai, China). GUS histochemical staining and activity were assessed using a GUS staining kit, following the manufacturer’s instructions (Coolaber Science & Technology Co., Ltd., Beijing, China).

### RNA-Seq analyses

Total RNA was extracted using TRIzol reagent (Invitrogen, Carlsbad, CA, USA). mRNAs were then purified using poly-T oligo-attached magnetic beads. Sequencing libraries were constructed using the NEBNext Ultra RNA Library Prep Kit for Illumina (#7530 L, NEB, USA) following the manufacturer’s instructions. After preparation, the libraries were sequenced on the Illumina HiSeq 4000 platform, producing 150-bp reads. Grape genome data and corresponding annotations were downloaded from the Grapevine Genome CRIBI Biotech website (http://genomes.cribi.unipd.it/grape/). Transcriptome assembly and quantification were performed using StringTie software (v. 2.1.3b). The expression levels of unigenes were quantified in terms of fragments per kilobase of transcript per million mapped reads (FPKM). The differentially expressed genes (DEGs) were screened using the following criteria: log2(fold change) ≥1 and false discovery rate (FDR) ≤ 0.05. Data analysis, including principal component analysis (PCA), volcano plots, and Kyoto Encyclopedia of Genes and Genomes (KEGG) enrichment analysis, was conducted using R software (v. 3.5.0) and BLASTALL, respectively.

### Targeted metabolomics analysis of phenolic compounds

Metabolite extraction, analysis, and determination were performed by OE Biotech Co., Ltd. (Shanghai, China). Briefly, lyophilized grape seeds were ground using a mixer mill (MM 400, Retsch). Metabolites were then extracted using 400 μl of chloroform and 600 μl of 70% methanol, followed by centrifugation and filtration. The resulting filtrate was analyzed using an ultraperformance liquid chromatography–electrospray ionization–tandem mass spectrometry (UPLC–ESI–MS/MS) system, which included an ExionLC UPLC and a QTRAP 6500+ mass spectrometer (AB SCIEX, Darmstadt, Germany). The UPLC and MS conditions were set according to the methods of our previous study [47]. The quantification of phenolic compounds was performed using an external calibration curve made with different phenolic compound standards. Differentially accumulated metabolites (DAMs) were considered based on fold change (FC) ≥ 2 and *P*-value ≤ 0.005.

### DNA affinity purification sequencing

For DNA affinity purification sequencing (DAP-seq), genomic DNA from grapes was extracted, purified, and fragmented into ~200-bp segments to construct a genomic DNA (gDNA) library. The CDS of *VvMYB14* was cloned into the pFN19K HaloTag vector. Purification of VvMYB14 and enrichment of its DNA targets were conducted by Bluescape Biotechnology Co. (Baoding, China). Clean reads were mapped to the grape genome by using Bowtie2 software [48]. *VvMYB14* binding peaks were identified using model-based analysis for ChIP-Seq (MACS2) software, with a peak defined by a *q* value of <0.05 [49].

### Real-time quantitative polymerase chain reaction

Total RNA was extracted using an RNAprep Pure Plant Kit (Tiangen, Beijing, China). The extracted RNA was then reverse-transcribed into complementary DNA by using the Hiscript Q RT SuperMix (Vazyme, Nanjing, China). Quantitative PCR (qPCR) was performed using ChamQ SYBR qPCR Master Mix (Vazyme, Nanjing, China) on an ABI7500 real-time quantitative PCR (qRT-PCR) instrument (ABI, MA, USA). The primers used for the qRT-PCR are listed in Table S1.

### Statistical analysis

Statistical analysis was performed using SPSS (V19.0) Statistics software. The significance of differences was determined by one-way analysis of variance (ANOVA) (*P* < 0.05) followed by Tukey’s test. Student’s *t*-test with a two-tailed distribution was used to compare two sample groups.

## Supplementary Material

Web_Material_uhaf061

## Data Availability

The full RNA-seq and DAP-seq data have been submitted to the Sequence Read Archive (SRA) of the NCBI under BioSample accession PRJNA1151140 and PRJNA1151207.

## References

[1] Yilmaz Y, Göksel Z, Erdoğan SS. et al. Antioxidant activity and phenolic content of seed, skin and pulp parts of 22 grape (Vitis vinifera L.) cultivars (4 common and 18 registered or candidate for registration). J Food Process Preserv. 2015;39:1682–91

[2] Hanlin RL, Kelm MA, Wilkinson KL. et al. Detailed characterization of Proanthocyanidins in skin, seeds, and wine of shiraz and cabernet sauvignon wine grapes (Vitis vinifera). J Agric Food Chem. 2011;59:13265–76 22085086 10.1021/jf203466u

[3] Xu L, Yue Q, Xiang G. et al. Melatonin promotes ripening of grape berry via increasing the levels of ABA, H2O2, and particularly ethylene. Hortic Res. 2018;5:41 30083356 10.1038/s41438-018-0045-y PMC6068098

[4] Ma W, Xu L, Gao S. et al. Melatonin alters the secondary metabolite profile of grape berry skin by promoting VvMYB14-mediated ethylene biosynthesis. Hortic Res. 2021;8:43 33642583 10.1038/s41438-021-00478-2 PMC7917092

[5] Xu L, Yue Q, Bian F. et al. Melatonin enhances Phenolics accumulation partially via ethylene signaling and resulted in high antioxidant capacity in grape berries. Front Plant Sci. 2017;8:1426 28868058 10.3389/fpls.2017.01426 PMC5563355

[6] Zhang X, Ma W, Guan X. et al. VvMYB14 participates in melatonin-induced proanthocyanidin biosynthesis by upregulating expression of VvMYBPA1 and VvMYBPA2 in grape seeds. Hortic Res. 2023;10:274 10.1093/hr/uhac274 PMC10390852 37533674

[7] Tallapally M, Sadiq AS, Mehtab V. et al. GC-MS based targeted metabolomics approach for studying the variations of phenolic metabolites in artificially ripened banana fruits. LWT. 2020;130:109622

[8] Jie H, He P, Zhao L. et al. Molecular mechanisms regulating Phenylpropanoid metabolism in exogenously-sprayed ethylene forage ramie based on transcriptomic and Metabolomic analyses. Plan Theory. 2023;12:3899 10.3390/plants12223899 PMC10675582 38005796

[9] Pirrello J, Prasad BCN, Zhang W. et al. Functional analysis and binding affinity of tomato ethylene response factors provide insight on the molecular bases of plant differential responses to ethylene. BMC Plant Biol. 2012;12:190 23057995 10.1186/1471-2229-12-190 PMC3548740

[10] Liu M, Li Z, Zhang Y. et al. Role of ethylene response factors (ERFs) in fruit ripening. Food Qual Saf. 2020;4:15–20

[11] Huang Q, Sun M, Yuan T. et al. The AP2/ERF transcription factor SmERF1L1 regulates the biosynthesis of tanshinones and phenolic acids in salvia miltiorrhiza. Food Chem. 2019;274:368–75 30372953 10.1016/j.foodchem.2018.08.119

[12] Arc E, Sechet J, Corbineau F. et al. ABA crosstalk with ethylene and nitric oxide in seed dormancy and germination. Front Plant Sci. 2013;4:63 23531630 10.3389/fpls.2013.00063 PMC3607800

[13] Leubner G, Ayele BT, Izydorczyk MS. et al. Ethylene regulates post-germination seedling growth in wheat through spatial and temporal modulation of ABA/GA balance. J Exp Bot. 2020;71:1985–2004 31872216 10.1093/jxb/erz566 PMC7094081

[14] Yue P, Wang Y, Bu H. et al. Ethylene promotes IAA reduction through PuERFs-activated PuGH3.1 during fruit ripening in pear (Pyrus ussuriensis). Postharvest Biol Technol. 2019;157:110955

[15] Shan S, Wang Z, Pu H. et al. DNA methylation mediated by melatonin was involved in ethylene signal transmission and ripening of tomato fruit. Sci Hortic. 2022;291:110566

[16] Back K, Tan DX, Reiter RJ. Melatonin biosynthesis in plants: multiple pathways catalyze tryptophan to melatonin in the cytoplasm or chloroplasts. J Pineal Res. 2016;61:426–37 27600803 10.1111/jpi.12364

[17] Arnao MB, Hernández-Ruiz J. Melatonin: a new plant hormone and/or a plant master regulator? Trends Plant Sci. 2019;24:38–48 30446305 10.1016/j.tplants.2018.10.010

[18] Wang J, Zhang H, Hou J. et al. Metabolic profiling and molecular mechanisms underlying melatonin-induced secondary metabolism of postharvest goji berry (Lycium barbarum L.). Food Secur. 2023;12:4326 10.3390/foods12234326 PMC10706708 38231790

[19] Caspi Y, Pantazopoulou CK, Prompers JJ. et al. Why did glutamate, GABA, and melatonin become intercellular signalling molecules in plants? elife. 2023;12:e83361 37338964 10.7554/eLife.83361 PMC10281673

[20] Priest HD, Filichkin SA, Mockler TC. Cis-regulatory elements in plant cell signaling. Curr Opin Plant Biol. 2009;12:643–9 19717332 10.1016/j.pbi.2009.07.016

[21] Höll J, Vannozzi A, Czemmel S. et al. The R2R3-MYB transcription factors MYB14 and MYB15 regulate stilbene biosynthesis in Vitis vinifera. Plant Cell. 2013;25:4135–49 24151295 10.1105/tpc.113.117127 PMC3877794

[22] Mu H, Li Y, Yuan L. et al. MYB30 and MYB14 form a repressor–activator module with WRKY8 that controls stilbene biosynthesis in grapevine. Plant Cell. 2023;35:552–73 36255259 10.1093/plcell/koac308 PMC9806661

[23] Liu C, Jun JH, Dixon RA. MYB5 and MYB14 play pivotal roles in seed coat polymer biosynthesis in Medicago truncatula. Plant Physiol. 2014;165:1424–39 24948832 10.1104/pp.114.241877 PMC4119029

[24] Roldan MB, Cousins G, Fraser K. et al. Elevation of condensed tannins in the leaves of Ta-MYB14-1 white clover (Trifolium repens L.) outcrossed with high anthocyanin lines. J Agric Food Chem. 2019;68:2927–39 31241924 10.1021/acs.jafc.9b01185

[25] Sun LA, Zhang M, Ren J. et al. Reciprocity between abscisic acid and ethylene at the onset of berry ripening and after harvest. BMC Plant Biol. 2010;10:257 21092180 10.1186/1471-2229-10-257 PMC3095336

[26] Chervin C, el-Kereamy A, Roustan JP. et al. Ethylene seems required for the berry development and ripening in grape, a non-climacteric fruit. Plant Sci. 2004;167:1301–5

[27] Pan Y, Seymour GB, Lu C. et al. An ethylene response factor (ERF5) promoting adaptation to drought and salt tolerance in tomato. Plant Cell Rep. 2011;31:349–60 22038370 10.1007/s00299-011-1170-3

[28] Pan , Zheng J, Liu J. et al. Analysis of the ASMT gene family in pepper (Capsicum annuum L.): identification, phylogeny, and expression profiles. Int J Genomics. 2019;2019:1–11 10.1155/2019/7241096 PMC6466892 31065551

[29] Yu Y, Ni Y, Qiao T. et al. Overexpression of VvASMT1 from grapevine enhanced salt and osmotic stress tolerance in Nicotiana benthamiana. PLoS One. 2022;17:e0269028 35709203 10.1371/journal.pone.0269028 PMC9202941

[30] Fan S, Li Q, Feng S. et al. Melatonin maintains fruit quality and reduces anthracnose in postharvest papaya via enhancement of antioxidants and inhibition of pathogen development. Antioxidants. 2022;11:804 35624668 10.3390/antiox11050804 PMC9137572

[31] Jayarajan S, Sharma RR. Melatonin: a blooming biomolecule for postharvest management of perishable fruits and vegetables. Trends Food Sci Technol. 2021;116:318–28

[32] Mo R, Han G, Zhu Z. et al. The ethylene response factor ERF5 regulates anthocyanin biosynthesis in ‘Zijin’ mulberry fruits by interacting with MYBA and F3H genes. Int J Mol Sci. 2022;23:7615 35886963 10.3390/ijms23147615 PMC9318412

[33] Chang Y-j, Chen G-s, Yang G-y. et al. The PcERF5 promotes anthocyanin biosynthesis in red-fleshed pear (Pyrus communis) through both activating and interacting with PcMYB transcription factors. J Integr Agric. 2023;22:2687–704

[34] Sun C, Liu L, Wang L. et al. Melatonin: a master regulator of plant development and stress responses. J Integr Plant Biol. 2020;63:126–45 32678945 10.1111/jipb.12993

[35] Shelton D, Stranne M, Mikkelsen L. et al. Transcription factors of lotus: regulation of isoflavonoid biosynthesis requires coordinated changes in transcription factor activity. Plant Physiol. 2012;159:531–47 22529285 10.1104/pp.112.194753 PMC3375922

[36] Barros J, Dixon RA. Plant phenylalanine/tyrosine ammonia-lyases. Trends Plant Sci. 2020;25:66–79 31679994 10.1016/j.tplants.2019.09.011

[37] Huang J, Gu M, Lai Z. et al. Functional analysis of the Arabidopsis PAL gene family in plant growth, development, and response to environmental stress. Plant Physiol. 2010;153:1526–38 20566705 10.1104/pp.110.157370 PMC2923909

[38] Zhang X, Abrahan C, Colquhoun TA. et al. A proteolytic regulator controlling chalcone synthase stability and flavonoid biosynthesis in Arabidopsis. Plant Cell. 2017;29:1157–74 28446542 10.1105/tpc.16.00855 PMC5466025

[39] Wang P, Yu A, Ji X. et al. Transcriptome and metabolite integrated analysis reveals that exogenous ethylene controls berry ripening processes in grapevine. Food Res Int. 2022;155:111084 35400460 10.1016/j.foodres.2022.111084

[40] Sharafi Y, Jannatizadeh A, Fard JR. et al. Melatonin treatment delays senescence and improves antioxidant potential of sweet cherry fruits during cold storage. Sci Hortic. 2021;288:110304

[41] Bethke G, Unthan T, Uhrig JF. et al. Flg22 regulates the release of an ethylene response factor substrate from MAP kinase 6 in *Arabidopsis thaliana* via ethylene signaling. Proc Natl Acad Sci USA. 2009;106:8067–72 19416906 10.1073/pnas.0810206106 PMC2683104

[42] Lee HY, Back K. Mitogen-activated protein kinase pathways are required for melatonin-mediated defense responses in plants. J Pineal Res. 2016;60:327–35 26927635 10.1111/jpi.12314

[43] Yang Q, Peng Z, Ma W. et al. Melatonin functions in priming of stomatal immunity in Panax notoginseng and Arabidopsis thaliana. Plant Physiol. 2021;187:2837–51 34618091 10.1093/plphys/kiab419 PMC8644721

[44] Tucker ML, Xue P, Yang R. 1-Aminocyclopropane-1-carboxylic acid (ACC) concentration and ACC synthase expression in soybean roots, root tips, and soybean cyst nematode (Heterodera glycines)-infected roots. J Exp Bot. 2010;61:463–72 19861652 10.1093/jxb/erp317 PMC2803212

[45] Xu L, Xiang G, Sun Q. et al. Melatonin enhances salt tolerance by promoting MYB108A-mediated ethylene biosynthesis in grapevines. Hortic Res. 2019;6:114 31645968 10.1038/s41438-019-0197-4 PMC6804660

[46] Clough SJ, Bent AF. Floral dip: a simplified method for agrobacterium-mediated transformation of Arabidopsis thaliana. Plant J. 2008;16:735–43 10.1046/j.1365-313x.1998.00343.x 10069079

[47] Gao S, Wang F, Zhang X. et al. Characterization of anthocyanin and nonanthocyanidin phenolic compounds and/or their biosynthesis pathway in red-fleshed ‘Kanghong’ grape berries and their wine. Food Res Int. 2022;161:111789 36192939 10.1016/j.foodres.2022.111789

[48] Langmead B, Salzberg SL. Fast gapped-read alignment with bowtie 2. Nat Methods. 2012;9:357–9 22388286 10.1038/nmeth.1923 PMC3322381

[49] Yu G, Wang L-G, He Q-Y. ChIPseeker: an R/Bioconductor package for ChIP peak annotation, comparison and visualization. Bioinformatics. 2015;31:2382–3 25765347 10.1093/bioinformatics/btv145

