# Supplementary material for: Ethylene modulates the phenylpropanoid pathway by enhancing VvMYB14 expression via the ERF5-melatonin-ERF104 pathway in grape seeds
Source: Hortic Res. 2025 Feb 25;12(6):uhaf061. doi: 10.1093/hr/uhaf061 (PMC12017797; doi:10.1093/hr/uhaf061)
Supplement: Web_Material_uhaf061 [file web_material_uhaf061.zip › Figure S5.pdf]

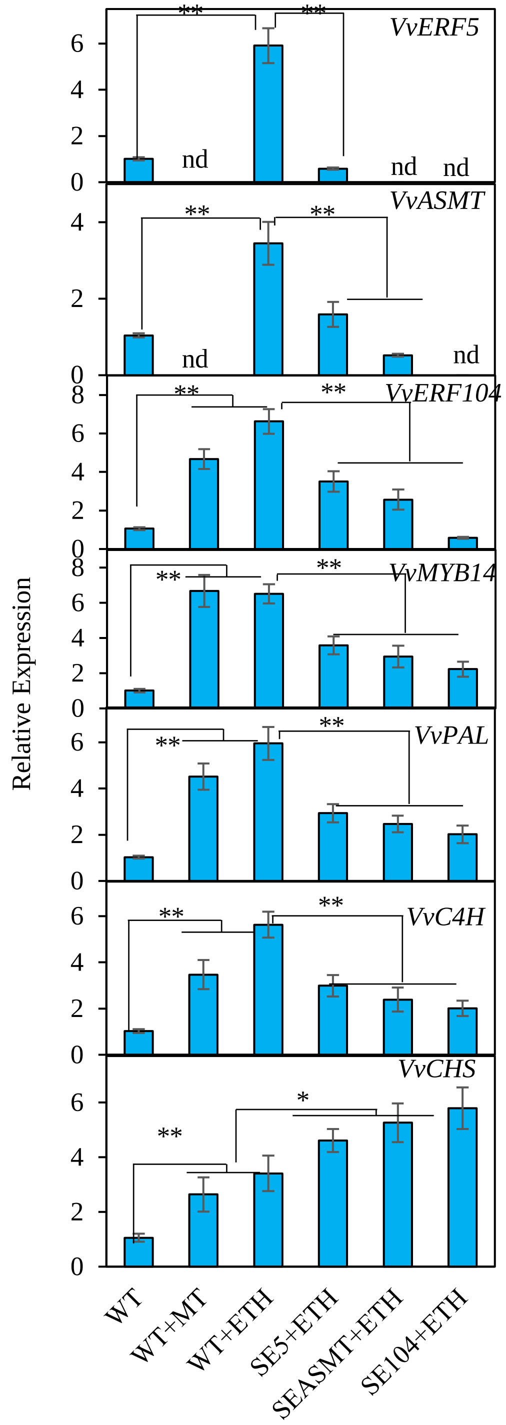

Supplemental Figure S5 Changes in gene expression in the control and ethephon- or melatonin-treated grape seeds with suppressed expression of *VvERF5*, *VvASMT*, and *VvERF104*.
