# Supplementary figures and images for: Ethylene modulates the phenylpropanoid pathway by enhancing VvMYB14 expression via the ERF5-melatonin-ERF104 pathway in grape seeds
Source: Hortic Res. 2025 Feb 25;12(6):uhaf061. doi: 10.1093/hr/uhaf061 (PMC12017797; doi:10.1093/hr/uhaf061)

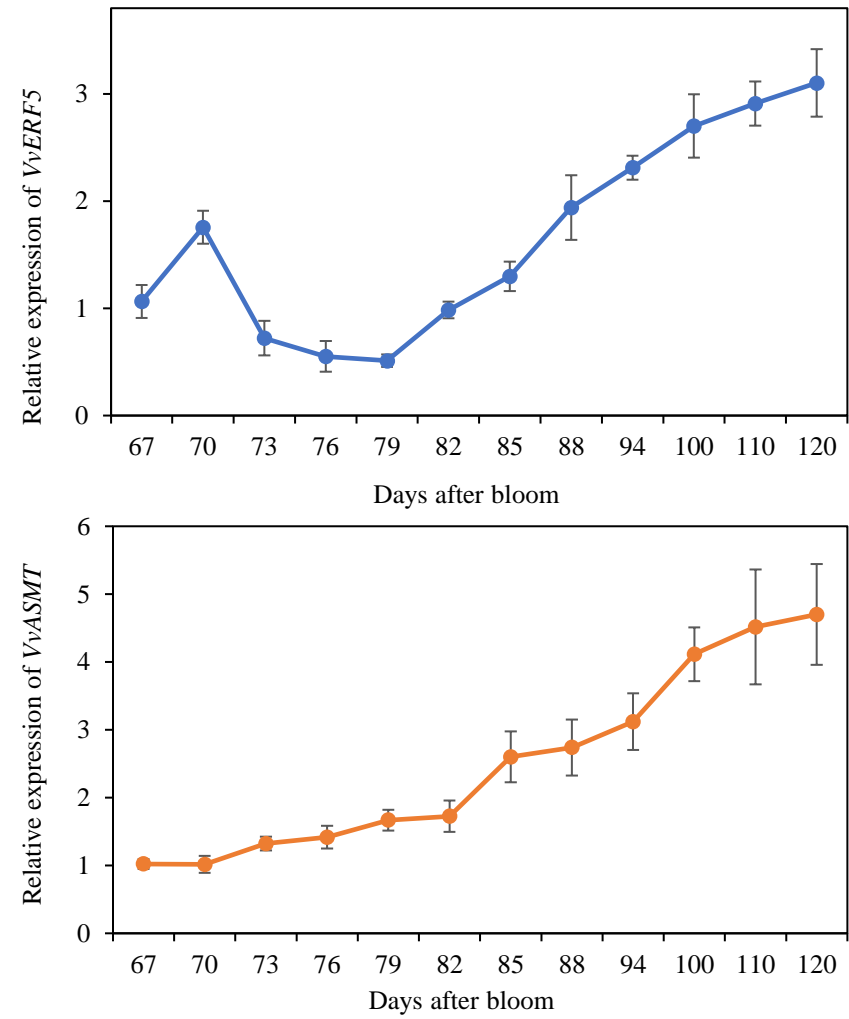

**Supplemental Figure S1** Expression patterns of *VvERF5* and *VvASMT* in grape seeds during ripening.

Supplement: Web_Material_uhaf061 [file web_material_uhaf061.zip › Figure S1.pdf]

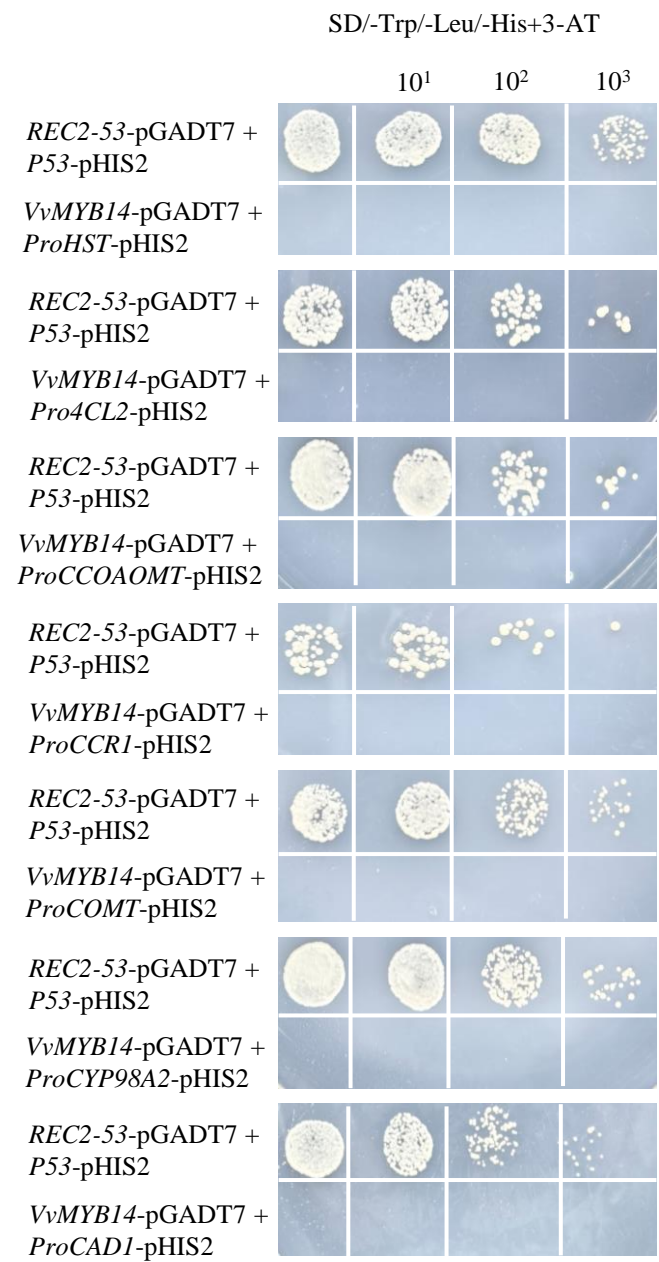

**Supplemental Figure S4** Y1H assays of VvMYB14 with seven possible target genes.

Supplement: Web_Material_uhaf061 [file web_material_uhaf061.zip › Figure S4.pdf]
